# Prevalence of neurocognitive disorder in Huntington’s disease using the Enroll-HD dataset

**DOI:** 10.3389/fneur.2023.1198145

**Published:** 2023-07-14

**Authors:** Luis A. Sierra, Clementina J. Ullman, Clara Baselga-Garriga, Sarbesh R. Pandeya, Samuel A. Frank, Simon Laganiere

**Affiliations:** ^1^Department of Neurology, Beth Israel Deaconess Medical Center, Boston, MA, United States; ^2^Harvard Medical School, Boston, MA, United States

**Keywords:** Huntington’s disease, cognition, neurocognitive disorders (NCD), pre-motor manifest, DSM-5

## Abstract

**Background:**

Cognitive decline in Huntington’s disease (HD) begins early in the disease course, however the reported prevalence and severity of cognitive impairment varies based on diagnostic approach. A Movement Disorders Society Task Force recently endorsed the use of standardized DSM-5-based criteria to diagnose neurocognitive disorder (NCD) in Huntington’s disease.

**Objectives:**

To determine the prevalence and severity of cognitive impairment across different stages of HD by applying NCD criteria (mild and major) to participant data from the Enroll-HD database.

**Methods:**

Enroll-HD participants were triaged into either premanifest (preHD), manifest or control groups. PreHD was further dichotomized into preHD near or preHD far based on predicted time to diagnosis using the scaled CAG-age product score (CAPs). Embedded cognitive performance and functional independence measures were used to determine prevalence of NCD (mild and major) for all groups.

**Results:**

Prevalence of NCD-mild was 25.2%–38.4% for manifest HD, 22.8%–47.3% for preHD near, 11.5%–25.1% for preHD far, and 8.8%–19.1% for controls. Prevalence of NCD-major was 21.1%–57.7% for manifest HD, 0.5%–16.3% for preHD near, 0.0%–4.5% for preHD far, and 0.0%–3.0% for controls.

**Conclusion:**

The prevalence of NCD in HD is elevated in preHD and demonstrates a sharp rise prior to diagnosis. In manifest HD, the vast majority of participants meet criteria for NCD. These findings are important for optimizing clinical care and/or anticipating the need for supportive services.

## Introduction

Huntington’s disease (HD) is a progressive disease characterized by a triad of symptoms, including movement abnormalities, cognitive impairment, and behavioral disturbances. While all three domains are typically involved, the diagnosis of manifest HD is confirmed by the presence of unequivocal motor signs. Although motor signs can be striking, cognitive impairment typically exerts a larger functional impact (1–3), appears earlier (4), and correlates with both early job termination (5) and impaired driving ability (6). Despite numerous studies demonstrating cognitive decline in both pre-motor manifest stage (preHD) and manifest HD (4), there is currently no standard approach for diagnosing cognitive impairment in this disease. As a result, the prevalence of cognitive impairment across different HD stages remains unclear. The lack of specific and standardized diagnostic criteria may obscure current or upcoming patient needs and has important implications for patients seeking workplace accommodations or disability (7).

In an effort to quantify cognitive impairment in HD, prior studies used the MCI model (8). Studies analyzing the PREDICT-HD database, for example, reported an MCI prevalence of 40% in preHD and 54% in prodromal participants (9). However, this framework was originally tailored to identify early symptoms of Alzheimer’s, prioritizing episodic memory loss and subjective decline (8). In contrast, HD patients often exhibit early deficits in executive functioning and are frequently unaware of their condition (i.e., anosognosia) (10–12). While the definition and use of the term of MCI has broadened since its original inception, and can include deficits in other domains, the definition of MCI in HD is not currently anchored to a single authoritative source. In recognition of potential limitation regarding terminology and criteria, the Movement Disorder Society Task Force for diagnostic classifications of HD (13) recently recommended using DSM-5-based criteria to classify HD-related cognitive impairment as either neurocognitive disorder mild (NCD-mild) or major (NCD-major) (Supplementary Figure S1) (14).

The primary potential benefits of using this framework are manifold: (1) it moves away from terminology that might still be associated primarily with Alzheimer’s disease, (2) it establishes a consistent standard across a variety of disciplines, including neurology, psychiatry, and neuropsychology, allowing for the synchronization and standardization of practices, (3) it encourages the use of quantitative thresholds, norms, and standard deviations to gauge the stage of impairment more accurately and (4) by linking the terminology and criteria to the DSM, this framework facilitates swift and substantial updates to current practices, ensuring that the field stays up-to-date with the latest definitions. Criteria for NCD-mild and NCD-major outline specific functional and cognitive performance thresholds for both diagnoses. NCD-mild is defined as a “modest” deviation from normative values (between 1–2 SD) and NCD-major requires both “substantial” deviation from the norm (>2 SD below the norm) as well as interference with independence in everyday activities in the absence of delirium. Although the prevalence of MCI has been reported across stages of HD, to our knowledge DSM-5-based criteria for NCD have not yet been applied to characterize this disease.

In this brief report, we used DSM-5 criteria for neurocognitive disorder to obtain an estimate of NCD prevalence in preHD (both near and far from predicted diagnosis) and manifest HD using Enroll-HD, the largest observational research platform in HD.^1^ We started by using the cognitive performance values and functional independence measures already present in the database but omitted MMSE and Trail Making Test due to concerns regarding floor/ceiling effects and non-normal distributions in the Enroll-HD dataset (15). It is important to emphasize that the cognitive assessments employed in this study are limited in their ability to fully capture all the cognitive impairments resulting from HD. Therefore, the results presented herein should be interpreted as a conservative estimate of NCD prevalence in this disease.

## Methods

### Participants

All participants analyzed in this study were selected from the Enroll-HD database and gave written consent to voluntarily participate in a yearly assessment as approved by local IRBs. Enroll-HD Periodic dataset (profile and enroll visit based specifically) was obtained through a data use agreement with cure Huntington’s disease initiative. Data included assessments of HD participants and healthy controls at their baseline and follow up visits between 2012–2022 (data cut 03/17/2022) across multiple approved US sites.^2^

### Data analysis

The Enroll-HD periodic dataset (PDS) obtained initially included 8,016 participants. Due to regulatory concerns related to General Data Protection Regulation (GDPR), only US-based data was transferred and analyzed. We applied category label filters of premanifest, manifest and controls. PreHD participants in the Enroll-HD database were defined, per protocol, as individuals without clinical features of manifest HD, as assessed and labeled by individual site investigators. Inclusion criteria for HD were: confirmed genetic diagnosis of mHTT with CAG repeats **≥**36, age between 18 and 78. Inclusion criteria for controls were: a label of either gene negative or family control and age between 18–78. The upper age cutoff of 78 was imposed by the age limit when converting to scaled scores (see below).

Each participant’s baseline visit was assessed along four dimensions of the Unified Huntington’s Disease Rating Scale (UHDRS^™^): functional independence (Independence Scale, IS); motor signs (UHDRS^™^ Total Motor Score, TMS), cognition (SCWT, SDMT, and FAS) and diagnostic confidence level (DCL). To prevent the influence of erroneous entries or outliers, we excluded data with implausible values based on score parameters. The following ranges (which represent minimal-maximal scores) were used for all cohorts: IS (0–100), TMS (0–124), and DCL (0–4). The range for cognitive testing on all participants was between 0 and maximal possible test score or set to a threshold that was above the highest observed performance in the dataset. Therefore, the ranges were the following: SDMT (0–110 correct responses), FAS (0–100 total words), and SWRT (0–149 correct responses for each subtest). The preHD cohort was further dichotomized into either preHD near or preHD far using a CAG-age product scaled (CAPs) score (CAPs = Age_0_ × (CAG − 33.66)/ 432.3326) of 0.85, which is equivalent to 7.59 years from predicted onset (16). In other words, preHD participants with CAPs >0.85 were included in the preHD near subgroup.

### Neuropsychological/functional assessments

Each participant’s cognition was assessed using SCWT (17), SDMT (18), and FAS (8). Performance on each cognitive test was converted from raw scores to scaled scores based on a combination of age and education per the following normative manuals: SCWT (19), FAS (20), SDMT (21). We then calculated the number of individuals impaired on each cognitive test using their scaled scores and a range of 1–2 standard deviations (SD) below the mean for NCD-mild, as recommended by DSM-5 (14). For NCD-major, participants needed to demonstrate “substantial” cognitive deficits that interfered with independence in everyday activities. Fortunately, the Enroll-HD database also includes the UHDRS^™^ Independence scale, which ranges from 0 to 100 with higher scores indicating better functioning. A score of 80 is defined as “pre-disease level of employment changes or ends; cannot perform household chores to pre-disease level, and may need help with finance” (22). Therefore, participants with both ≥2 SD below the mean on cognitive testing and (IS) ≤80 were classified as having NCD-major. Participants that met only 1 of 2 criteria for NCD-major were classified as NCD-mild.

### Statistical analysis

**Figure 1:**
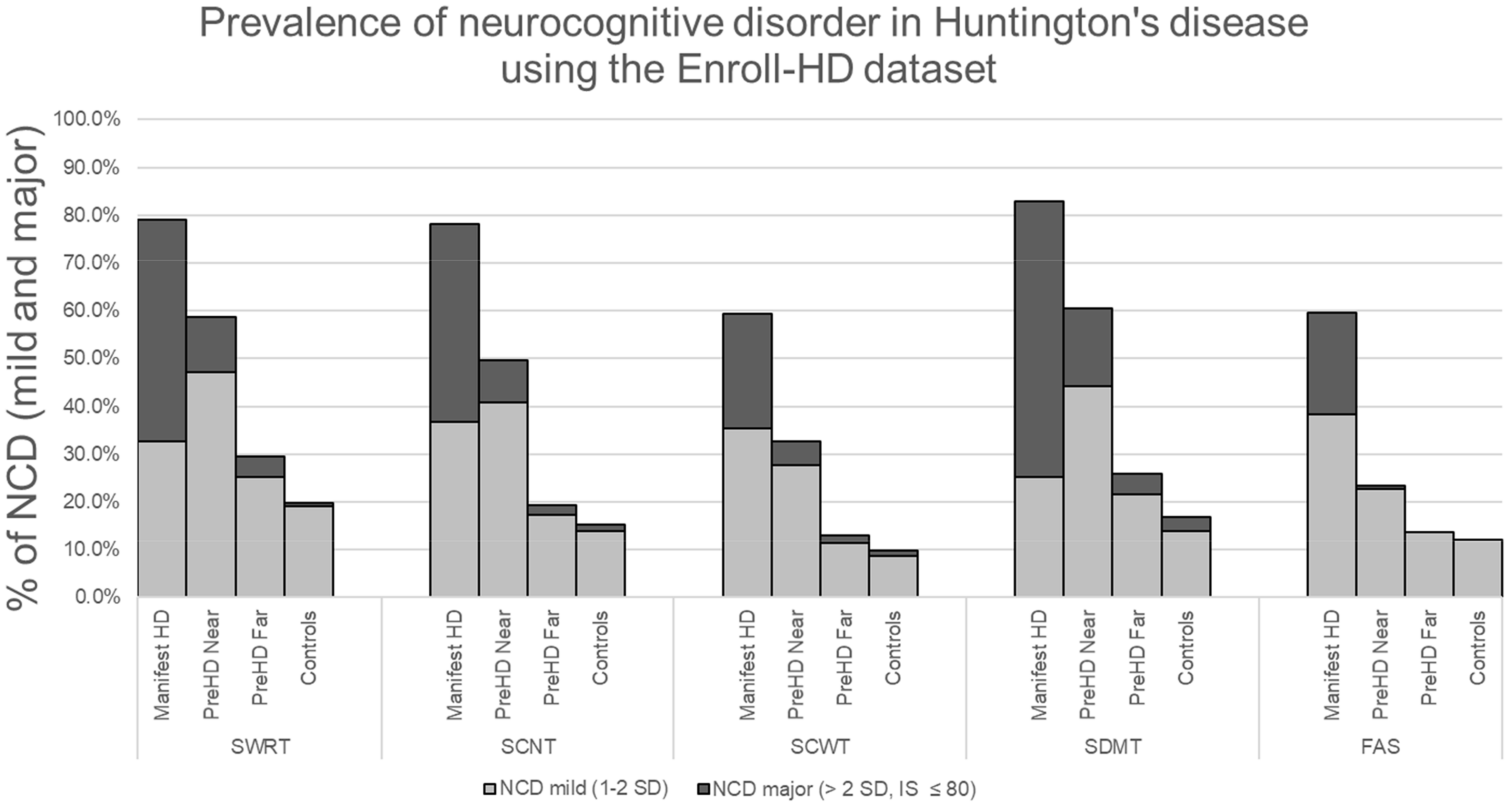
^*^Stroop word reading test (SWRT), Stroop color naming test (SCNT), Stroop color-word test, symbol digit modalities test (SDMT), verbal fluency (FAS), neurocognitive disorder (NCD).

All of our results, including the assessment of group means and standard deviations, data filtration and exclusion, were managed in R 4.2.1 (R Foundation for Statistical Computing, Vienna, Austria). To calculate the prevalence of NCD, we determined the number of participants in each subgroup (near, far, manifest and controls) who met criteria for NCD-mild and NCD-major (using performance on each cognitive assessment) and divided that number by each subgroup total. This resulted in a prevalence of NCD for each cognitive assessment (Figure 1). In other words, impairment on any one cognitive test implies impairment in at least one cognitive domain targeted by that test, which fulfills the requirement for NCD, as outlined by DSM-5 and MDS. NCD prevalence is therefore shown as a range, depending on the cognitive assessment used. Since the criteria can be fulfilled with an impairment in any one cognitive area, and it is impossible to cover all domains with the limited test battery, the prevalence of NCD is at least as high as the upper end of the reported range. Unpaired *T*-tests were used for all group comparisons except for sex, which was compared using 𝛸^2^ (Table 1).

**Table 1:** Demographics and cognitive testing.

Mean (SD)					*T*-test; *p*-values					
	Manifest (*n* = 1,967)	PreHD near (*n* = 601)	PreHD far (*n* = 1,070)	HC (*n* = 1,969)	PreHD near vs. manifest	PreHD far vs. manifest	Control vs. manifest	PreHD near vs. far	PreHD far vs. control	PreHD near vs. control
Age	51.40 (12.40)	47.48 (11.81)	36.29 (11.60)	47.40 (14.76)	^***^	^***^	^***^	^***^	^***^	0.903
Education (ISCED)	4.07 (1.02)	4.17 (0.99)	4.32 (0.90)	4.22 (0.95)	0.034	^***^	^***^	^***^	^***^	0.264
Sex (female)	51%	61%	64%	62%	^***^	^***^	^***^	0.190	0.254	0.634
CAG	43.90 (3.99)	43.34 (2.97)	41.76 (2.47)	—	0.002	^***^	—	^***^	—	—
TMS	30.20 (15.40)	6.00 (6.00)	2.00 (4.00)	1.73 (3.25)	^***^	^***^	^***^	^***^	0.044	^***^
TFC	9.48 (2.79)	12.00 (1.00)	13.00 (0.80)	12.90 (0.51)	^***^	^***^	^***^	^***^	^***^	^***^
Independence score	84.70 (12.30)	98.00 (5.00)	99.00 (3.00)	99.70 (1.63)	^***^	^***^	^***^	^***^	^***^	^***^
Stroop word reading	63.30 (19.70)	83.96 (18.36)	96.32 (17.11)	94.80 (16.46)	^***^	^***^	^***^	^***^	0.017	^***^
Stroop color naming	49.30 (15.06)	66.11 (15.03)	76.60 (14.10)	75.74 (13.86)	^***^	^***^	^***^	^***^	0.105	^***^
Stroop color-word (interference)	27.10 (10.50)	37.87 (10.94)	45.82 (10.90)	43.00 (10.75)	^***^	^***^	^***^	^***^	^***^	^***^
SDMT	28.80 (11.50)	43.50 (11.25)	53.83 (10.82)	51.07 (11.13)	^***^	^***^	^***^	^***^	^***^	^***^
FAS	25.80 (12.80)	37.48 (12.78)	42.04 (12.53)	42.36 (12.67)	^***^	^***^	^***^	^***^	0.503	^***^

(^***^p < 0.0001).

## Results

To maintain consistent sample size and simplify the analysis across all variables we removed any participant if they had any erroneous entry codes such as (9,998 or 9,997), blanks or outliers for any variable and then applied inclusion–exclusion criteria as noted in the methods. 2,409 participants were removed from the analysis (1,117 were removed because of erroneous entries or outliers and 1,292 due to exclusion criteria). This left a cohort of 5,610 participants that included 1,967 labeled as manifest HD, 601 as preHD near, 1,070 as preHD far, and 1,969 controls. The mean ± SD for age, education (International Standard Classification of Education, ISCED), sex, CAG, TMS, TFC, and IS can be seen on Table 1. Prevalence of NCD-mild exhibited a range, depending on cognitive assessment, of 25.2%–38.4% for manifest HD, 22.8%–47.3% for preHD near, 11.5%–25.1% for preHD far, and 8.8%–19.1% for controls. Prevalence of NCD-major was 21.1%–57.7% for manifest HD, 0.5%–16.3% for preHD near, 0.0%–4.5% for preHD far, and 0.0%–3.0% for controls. The total prevalence of NCD (mild + major) in HD had the following ranges: manifest HD: 59.4%–82.9%; preHD near: 23.3%–60.6%; preHD far: 13.0%–29.5% and controls: 9.9%–19.9%. Table 1 shows the mean scores ± SD for each cognitive test. The number and percent of participants with both NCD-mild and NCD-major is shown on Table 2 and Figure 1. Group comparisons are shown on Table 1.

**Table 2:** Cognitive testing performance.

		Mean (SD)			NCD mild: participants 1–2 SD below normative performance, *n* (%)				NCD major: participants >2 SD below normative performance and IS ≤80, *n* (%)			
	Manifest (*n* = 1,967)	PreHD near (*n* = 601)	PreHD far (*n* = 1,070)	Control (*n* = 1,969)	Manifest	PreHD near	PreHD far	Control	Manifest	PreHD near	PreHD far	Control
Stroop word reading	63.30 (19.70)	83.96 (18.36)	96.32 (17.11)	94.80 (16.46)	642 (32.6%)	284 (47.3%)	269 (25.1%)	376 (19.1%)	912 (46.4)	68 (11.3%)	47 (4.4%)	15 (0.8%)
Stroop color naming	49.30 (15.06)	66.11 (15.03)	76.60 (14.10)	75.74 (13.86)	721 (36.7%)	245 (40.8%)	185 (17.3%)	274 (13.9%)	817 (41.5%)	53 (8.8%)	21 (2.0%)	27 (1.4%)
Stroop color-word (interference)	27.10 (10.50)	37.87 (10.94)	45.82 (10.90)	43.00 (10.75)	697 (35.4%)	166 (27.6%)	123 (11.5%)	173 (8.8%)	472 (24.0%)	31 (5.2%)	16 (1.5%)	21 (1.1%)
SDMT	28.80 (11.50)	43.50 (11.25)	53.83 (10.82)	51.07 (11.13)	495 (25.2)	266 (44.3%)	230 (21.5%)	272 (13.8%)	1,135 (57.7%)	98 (16.3%)	48 (4.5%)	59 (3.0%)
FAS	25.80 (12.80)	37.48 (12.78)	42.04 (12.53)	42.36 (12.67)	756 (38.4%)	137 (22.8%)	147 (13.7%)	240 (12.2%)	416 (21.1%)	3 (0.5%)	0 (0.0%)	0 (0.0%)

## Discussion

In this study, we investigated the prevalence of neurocognitive disorder at various stages of Huntington’s disease using Enroll-HD, the largest publicly available HD database. Using an approach recommended by both the Movement Disorder Society and DSM-5, a conservative estimate for the prevalence of NCD in HD is 82.9% for manifest HD, 60.6% for preHD (<7.6 years from diagnosis), 29.5% for preHD far (>7.6 years from diagnosis), and 19.9% for control. These data demonstrate an elevated rate of NCD across many stages of HD and underline the acceleration of cognitive decline as preHD participants approach the manifest stage (13). These results are consistent with reports of MCI in preHD of between 40%–54% (8, 9) and extend these findings using the largest available HD research platform.

The high prevalence of cognitive impairment using NCD criteria on tests already embedded within the Enroll-HD study highlights important issues. First, NCD is likely under-recognized and/or underreported in clinical settings, especially in premanifest stages. As a result, opportunities for documenting NCD early in the disease course may be overlooked, which can lead to difficulty establishing a timeline for disability (23). Second, given the high incidence of symptom denial or anosognosia in HD (10–12), a diagnosis of NCD-mild, in particular, could alert the physician, patient or family to the presence of early deficits, which could accelerate the implementation of important safety measures (e.g., driving recommendations) or workplace accommodations.

There are some limitations to our study. The data acquired from Enroll-HD was limited to participants in the United States and excluded participants without genetic testing. These factors could have affected the overall generalizability of our results. Investigators across numerous different sites were responsible for labeling each HD participant as either pre-manifest and manifest. The theoretical use of varied or idiosyncratic thresholds or definitions for these labels could have influenced the result. Reassuringly, however, the rates of NCD-major are relatively small in the preHD far group; they also progressively increase with each stage. Overall, variability in participant labeling was likely minimized by the large sample size. DSM-5 criteria use the terms “modest” and “substantial” to describe the severity of cognitive impairment associated with mild and major NCD, respectively (14). These terms are associated with inherently arbitrary cutoffs. We used a performance cutoff of >1 SD for modest and >2 SD for substantial impairment, which is consistent with prior literature (24) and DSM-5 guidelines (14). The manifest HD group was, on average, older, included more males and had slightly fewer years of education than the other groups. These three factors could have led to slightly worse performance on cognitive testing and increased NCD prevalence in the manifest group. At the same time, these confounding effects should have been minimized by the use of scores that were scaled by age and education. Variables unaccounted for in the analysis, such as depression, anxiety, medication effects, testing environment or the presence of other preexisting developmental or neurological comorbidities could have influenced cognitive performance, future studies investigating the reliability of these results using longitudinal analyses of NCD in Enroll-HD would mitigate some of these concerns. The Enroll-HD cognitive tests used in this analysis are admittedly limited and likely insufficient to characterize the full spectrum of neurocognitive dysfunction in HD. In this regard, however, our results likely represent an underestimate, rather than an overestimate, of the actual prevalence of NCD at various stages of HD. Further research should continue to evaluate the sensitivity of other cognitive assessments for capturing cognitive decline in HD.

In conclusion, this report assessed the prevalence of neurocognitive disorder (mild and major) in premanifest HD (both near and far from predicted diagnosis) and manifest HD by applying DSM-5 criteria to cognitive performance measures embedded in the Enroll-HD database. NCD prevalence is elevated early in the disease course and appears to accelerate in the premanifest stage prior to predicted diagnosis. By the manifest stage, the vast majority of patients meet criteria for NCD. These findings have important research and clinical implications for the HD community.

## Data availability statement

Publicly available datasets were analyzed in this study. This data can be found at: https://www.enroll-hd.org/for-researchers/pds-data-explorer/.

## Ethics statement

The studies involving human participants were reviewed and approved by BIDMC Institutional Review Board. The patients/participants provided their written informed consent to participate in this study.

## Author contributions

LS and SL: conception, organization, execution, writing of the first draft, and review and critique. CU: conception, organization, execution, and review and critique. CB-G: writing of the first draft and review and critique. SP: design and execution. SF: conception, organization, and review and critique. All authors contributed to the article and approved the submitted version.

## Conflict of interest

The authors declare that the research was conducted in the absence of any commercial or financial relationships that could be construed as a potential conflict of interest.

## Publisher’s note

All claims expressed in this article are solely those of the authors and do not necessarily represent those of their affiliated organizations, or those of the publisher, the editors and the reviewers. Any product that may be evaluated in this article, or claim that may be made by its manufacturer, is not guaranteed or endorsed by the publisher.

## Footnotes

^1^https://www.enroll-hd.org/

^2^https://www.enroll-hd.org/for-researchers/pds-data-explorer/
