# Supplementary material for: Prevalence of neurocognitive disorder in Huntington’s disease using the Enroll-HD dataset
Source: Front Neurol. 2023 Jul 14;14:1198145. doi: 10.3389/fneur.2023.1198145 (PMC10375015; doi:10.3389/fneur.2023.1198145)
Supplement: Supplementary file 1 [file Table_1.DOCX]

**Supplemental Figure 1**

NEUROCOGNITIVE DISORDER

MAJOR

A.  Evidence of significant cognitive decline from a previous level of performance in one or more cognitive domains (complex attention, executive function, learning and memory, language, perceptual-motor, or social cognition) based on:

1.   Concern of the individual, a knowledgeable informant, or the clinician that there has been a mild decline in cognitive function; and

2.   A substantial impairment in cognitive performance, preferably documented by standardized neuropsychological testing or, in its absence, another quantified clinical assessment

B.  The cognitive deficits interfere with independence in everyday activities. (i.e. - at a minimum, requiring assistance with complex instrumental activities of daily living such as paying bills or managing medications)

C.  The cognitive deficits do not occur exclusively in the context of a delirium.

MILD

A.  Evidence of modest cognitive decline from a previous level of performance in one or more cognitive domains (complex attention, executive function, learning and memory, language, perceptual-motor, or social cognition) based on:

1.   Concern of the individual, a knowledgeable informant, or the clinician that there has been a mild decline in cognitive function; and

2.   A modest impairment in cognitive performance, preferably documented by standardized neuropsychological testing or, in its absence, another quantified clinical assessment

B.   The cognitive deficits do not interfere with capacity for independence in everyday      activities. (i.e. - complex instrumental activities of daily living such as paying bills or managing medications are preserved, but greater effort, compensatory strategies, or accommodation may be required).

C.   The cognitive deficits do not occur exclusively in the context of a delirium
